# Current Knowledge on Correlations Between Highly Prevalent Dental Conditions and Chronic Diseases: An Umbrella Review

**DOI:** 10.5888/pcd16.180641

**Published:** 2019-09-26

**Authors:** Max W. Seitz, Stefan Listl, Andreas Bartols, Ingrid Schubert, Katja Blaschke, Christian Haux, Marieke M. Van Der Zande

**Affiliations:** 1 University of Heidelberg, Institute of Medical Biometry and Informatics, Heidelberg, Germany; 2 Section for Translational Health Economics, Department of Conservative Dentistry, Heidelberg University, Heidelberg, Germany; 3 Radboud University Medical Center, Radboud Institute for Health Sciences, Department of Dentistry — Quality and Safety of Oral Healthcare, Nijmegen, The Netherlands; 4 Dental Academy for Continuing Professional Development, Karlsruhe, Germany; 5 Christian-Albrechts-University Kiel, Clinic for Conservative Dentistry and Periodontology, Kiel, Germany; 6 PMV Research Group, Faculty of Medicine and University Hospital Cologne, University of Cologne, Cologne, Germany

## Abstract

**Introduction:**

Studies have investigated the relationships between chronic systemic and dental conditions, but it remains unclear how such knowledge can be used in clinical practice. In this article, we provide an overview of existing systematic reviews, identifying and evaluating the most frequently reported dental–chronic disease correlations and common risk factors.

**Methods:**

We conducted a systematic review of existing systematic reviews (umbrella review) published between 1995 and 2017 and indexed in 4 databases. We focused on the 3 most prevalent dental conditions and 10 chronic systemic diseases with the highest burden of disease in Germany. Two independent reviewers assessed all articles for eligibility and methodologic quality using the AMSTAR criteria and extracted data from the included studies.

**Results:**

Of the initially identified 1,249 systematic reviews, 32 were included for qualitative synthesis. The dental condition with most frequently observed correlations to chronic systemic diseases was periodontitis. The chronic systemic disease with the most frequently observed correlations with a dental condition was type 2 diabetes mellitus (T2DM). Most dental–chronic disease correlations were found between periodontitis and T2DM and periodontitis and cardiovascular disease. Frequently reported common risk factors were smoking, age, sex, and overweight. Using the AMSTAR criteria, 2 studies were assessed as low quality, 26 studies as moderate quality, and 4 studies as high quality.

**Conclusion:**

The quality of included systematic reviews was heterogeneous. The most frequently reported correlations were found for periodontitis with T2DM and for periodontitis with cardiovascular disease. However, the strength of evidence for these and other disease correlations is limited, and the evidence to assess the causality of these disease correlations remains unclear. Future research should focus on the causality of disease links in order to provide more decisive evidence with respect to the design of intersectoral care processes.

**Summary**

**What is already known on this topic?**

Substantive evidence supports a correlation between dental conditions and chronic systemic diseases.

**What is added by this report?**

We provide an overview of systematic reviews reporting on correlations between dental conditions and chronic diseases with an assessment of the evidence and extent of correlation.

**What are the implications for public health practice?**

There is a need for more awareness about 1) existing evidence on correlations between dental conditions and chronic systemic diseases, 2) potential opportunities for better medical–dental integration in the delivery of care, and 3) the need for future research about potentially causal links between dental conditions and chronic diseases.

## Introduction

Human life expectancy has been increasing for many years (1). However, as life expectancy increases, so does the prevalence of chronic diseases within the population (2). Treatment of chronic diseases frequently takes place in highly specialized disciplines (3). However, chronic conditions often emerge, develop, and occur in parallel with other illnesses (4), and with each chronic condition life expectancy again decreases (5). Because of the high likelihood of patients with chronic conditions developing additional diseases, scientific study of the correlations between diseases is necessary.

The medical scope of such correlations often exceeds the boundaries of medical disciplines. An example of this is the correlation between dental conditions and other noncommunicable diseases, which have been investigated in many scientific publications and in previous empirical literature (6). In the past decades, however, dental care and primary medical care have largely evolved separately. Addressing the links between dental and other chronic conditions can improve health care and prevention of chronic conditions (7), in particular identifying appropriate and necessary areas for inter-professional cooperation between general medical and dental professionals (7).

Many systematic reviews (SRs) to estimate the extent of dental–chronic disease correlations have been conducted for specific dental conditions and chronic systemic diseases, but a systematic overview to provide information about the extent to which there is decisive evidence with respect to the design of intersectoral care processes does not exist so far. The aim of this study was to conduct an umbrella review to provide an overview of the most recent evidence from SRs about interdependencies between dental conditions and chronic systemic diseases. The underlying research question was, “What is the current state of knowledge concerning possible relationships between dental conditions and chronic systemic diseases?” The umbrella review aimed to identify potential intervention points for inter-professional cooperation, including evidence on 1) correlations between highly prevalent dental conditions and chronic systemic diseases, 2) common risk factors, and 3) how dental conditions cause chronic diseases and vice versa. 

## Methods

This study was conducted as part of a project aiming to improve intersectoral care between dentists and general practitioners (8). The results of this literature review will be combined with an analysis of claims data and patient reported measures into a decision support system (DSS). The DSS targets links between dental conditions and chronic systemic diseases managed in dental and primary care in Germany. The umbrella review focused on the chronic systemic diseases and dental conditions with the highest prevalence in Germany (9). The prevalence of these conditions in Germany is comparable to that of other Western European countries (10).

### Data sources

The scope of the review was defined using the PICO structure (11). The target population was defined as patients with a combination of 1) a chronic systemic disease and 2) 1 of the 3 dental conditions with the highest burden of disease: periodontitis (*International Classification of Diseases, 10th Revision* [ICD-10] K05), dental caries (ICD-10: K02.0), and tooth loss (ICD-10: K08.1) (12,13). There were no restrictions with respect to the type of (comparative) interventions or the (dental) health outcomes considered.

The search strategy was jointly developed by the authors (M.S., S.L., C.H., M.vdZ.) and sense-checked by 2 experts in dental and primary care and pharmacology. A librarian specializing in SRs reviewed the search strategy. For dental conditions the search terms were adjusted from the study by Haag et al (14).

The applied search strategy we used for PubMed is as follows:

(“Dental Caries”[Mesh] OR “Periodontal Diseases”[Mesh] OR “Mouth, Edentulous”[Mesh]
OR ((tooth[tiab] OR teeth[tiab] OR dental) AND (caries[tiab] OR carious[tiab] OR decay*[tiab] OR lesion*[tiab]))
OR “root caries”[tiab] OR “root decay”[tiab] OR “DMF Index”[tiab] OR “DMFT”[tiab] OR “DMFS”[tiab]
OR periodontal disease*[tiab] OR periodontitis[tiab] OR periodontal pocket*[tiab] OR periodontology[tiab]
OR “periodontal therapy”[tiab] OR periodontal treatment*[tiab] OR “periodontics”[tiab] OR “tooth loss”[tiab]
OR “number of teeth”[tiab] OR “shortened dental arch”[tiab] OR “functional dentition”[tiab] OR edentul*[tiab]
OR “missing teeth”[tiab] OR “missing tooth”[tiab] OR prosthodontics[tiab])
AND (“Chronic Disease”[Mesh] OR “Disease Progression”[Mesh] OR “Cardiovascular Diseases”[Mesh]
OR “Diabetes Mellitus”[Mesh] OR “Lung Diseases, Obstructive”[Mesh] OR “Pneumonia”[Mesh]
OR “Arthritis, Rheumatoid”[Mesh] OR ((disease[tiab] OR diseases[tiab] OR condition[tiab]
OR illness[tiab] OR ill[tiab] OR diseased[tiab]) AND (chronic[tiab] OR chronically[tiab]
OR systemic[tiab] OR cardiovascular[tiab] OR cerebrovascular[tiab])) OR “diabetes mellitus”[tiab]
OR “glycemic control”[tiab] OR diabetes[tiab] OR hyperglycemia[tiab] OR stroke[tiab] OR “cerebral ischemia”[tiab]
OR bronchitis[tiab] OR “pulmonary disease”[tiab] OR pneumonia[tiab] OR “rheumatoid arthritis”[tiab] OR Aspiration[tiab])
AND systematic[sb]
NOT (“animals”[Mesh] NOT “humans”[Mesh])

The search strategy was adapted for the searches in Embase, Cochrane, and LILACS. More details can be found here: https://doi.org/10.11588/data/ORTPJN.

Because of the multiple existing definitions for periodontitis, the search strategy was developed liberally to include a broad definition of periodontal disease. In addition, chronic diseases were addressed under various definitions (15). We used the term to refer to the definition by the World Health Organization (WHO): “Noncommunicable diseases . . . also known as chronic diseases, are not passed from person to person. They are of long duration and generally slow progression” (16). To further refine the search and include results on specific chronic diseases, diabetes (ICD-10: E10-E14), cardiovascular disease (CVD) (ICD-10: I20-I25), and chronic respiratory diseases (ICD-10: J40-J47) were prioritized as highly prevalent chronic conditions (9). Additionally (in their initial and moderate phase), they can be primarily detected and comprehensively managed in primary care.

A comprehensive literature search was performed on the PubMed, Embase, Cochrane, and LILACS databases in October 2017, including articles published up to 2017. EndNote version X8.1 was used for reference management (Clarivate Analytics). Duplicate references were excluded before article assessment. Two reviewers (M.S. and M.vdZ.) screened the title and abstract of all articles independently, excluding all records that did not meet the inclusion criteria. Based on the results of title and abstract screening, the inclusion criteria for the full-text screening were extended for the 10 chronic systemic diseases with the highest burden of disease. Those were defined as diseases that cause the most combined death and disability in Germany (9): ischemic heart disease, low back and neck pain, sensory organ diseases, cerebrovascular disease, lung cancer, Alzheimer disease, skin diseases, diabetes, chronic obstructive pulmonary disease (COPD), and migraine. The full text for all remaining articles was retrieved where available. In a second round, the articles were assessed by full text, using the adapted inclusion and exclusion criteria. Differences in assessment were discussed by the 2 reviewers, and in case of disagreements, a third reviewer (S.L.) made the final decision to include or exclude the article. The data from the remaining full-text articles were then extracted and the quality of the articles assessed.

### Study selection

After the database searches were conducted, all potential articles were aggregated in EndNote. The articles were screened by title and abstract for relevance. To ascertain interrater reliability, a calibration between the reviewers was conducted. The decision for inclusion or exclusion by both reviewers was compared for the first 100 screened articles and agreement was calculated by means of the Kappa value (17). Discrepancies were solved by an open discussion between the reviewers. If no consent could be reached, the third reviewer (S.L.) made the final decision.

Study inclusion criteria were 1) must be published in English; 2) must be an SR, a meta-analysis, or an umbrella review; 3) must be on patients with one of the predefined dental conditions (periodontitis, dental caries, or tooth loss) and a chronic systemic disease; and 4) must report on the link between the diseases. Studies were excluded if they 1) did not meet the inclusion criteria; 2) reported exclusively on children or animals; 3) did not report precisely the underlying search strategy; 4) contained no clear criteria for inclusion and exclusion of articles; 5) had not searched multiple databases; 6) did not include original studies; 7) reported on the same study as another included systematic review; 8) were included in another study that was already included; and 9) reported exclusively on a) a confounder and a dental condition but not a chronic systemic disease or b) a confounder and a chronic systemic disease but not a dental condition. The complete list of articles excluded in the full text screening, with reason for exclusion, can be found here: https://doi.org/10.11588/data/ORTPJN.

### Data extraction

The data from the articles included for qualitative synthesis were independently extracted by the 2 reviewers by using a standardized data collection form. Quantitative synthesis was not possible, because the included systematic reviews reported on correlations between various combinations of diseases. The 2 reviewers independently assessed the methodologic quality of the identified studies using the AMSTAR 11-point checklist (18), a measurement tool for assessing the quality of reporting of systematic reviews. Studies were designated as low quality if they met 0 to 3 criteria, moderate quality if they met 4 to 7 criteria, and high quality if they met 8 to 10 criteria. Discrepancies were discussed between the reviewers until agreement was reached on all items (Table 1). After this, the remaining articles were assessed.

**Table 1:** Results of the Quality Assessment for Included Systematic Reviews Using AMSTAR Checklist, Systematic Umbrella Review of Correlation Between Prevalent Dental Conditions and Chronic Diseases, 1995–2017

Study (year)	1. A Priori Design	2. Duplicate Selection	3. Literature Search	4. Status of Publication	5. List of Studies	6. Characteristics of Studies	7. Quality of Studies	8. Scientific Quality	9. Appropriate Methods	10. Likelihood of Bias	11. Conflict of Interest	Score
Abduljabbar, Javed et al (2017) (19)	0	1	1	0	1	1	1	1	1	0	0	7
Abduljabbar, Vohra et al (2017) (20)	1	1	1	1	1	1	1	1	1	0	0	9
Al-Hamoudi (2017) (21)	0	0	1	0	0	1	1	0	1	0	0	4
Azarpazhooh and Leake (2006) (22)	0	0	1	0	1	1	1	0	0	0	0	4
Batista et al (2011) (23)	0	0	1	1	1	1	0	0	0	0	0	4
Botero et al (2016) (24)	1	1	1	1	0	1	1	1	1	1	0	9
Dai et al (2015) (25)	0	1	1	0	1	1	1	0	1	1	0	7
D'Aiuto et al (2017) (26)	0	1	1	0	0	1	0	0	1	0	0	4
D'Aiuto et al (2013) (27)	0	1	1	0	0	1	1	0	0	0	1	5
Dietrich et al (2017) (28)	0	0	1	0	0	1	1	0	0	0	0	3
Faggion et al (2016) (29)	0	1	1	0	1	1	1	1	1	1	1	9
Hasuike et al (2017) (30)	1	0	1	0	0	1	1	1	1	0	1	7
Kelly et al (2013) (31)	0	1	1	0	1	0	1	1	1	0	1	7
Kothari et al (2017) (32)	0	1	1	1	0	1	0	0	1	0	0	5
Lafon et al (2014) (33)	0	1	1	0	0	1	1	0	1	0	0	5
Lam et al (2011) (34)	0	1	1	0	0	1	0	0	0	0	0	3
Leira et al (2017) (35)	1	1	1	0	0	1	1	1	1	0	0	7
Leng et al (2015) (36)	1	0	1	0	0	1	0	0	1	1	0	5
Li et al (2014) (37)	1	1	1	1	1	1	1	1	0	1	1	10
Lira et al (2017) (38)	0	1	1	1	1	1	1	0	1	0	0	7
Martin-Cabezas et al (2016) (39)	0	1	1	0	0	1	0	0	1	0	0	4
Mauri-Obradors et al (2017) (40)	0	1	1	0	0	1	1	0	0	0	0	4
Orlandi et al (2014) (41)	0	1	1	1	0	1	1	0	1	1	0	7
Sanchez et al (2017) (42)	0	0	1	1	0	1	1	0	0	0	0	4
Schmitt et al (2015) (43)	0	1	1	1	1	1	1	0	1	0	0	7
Teeuw et al (2014) (44)	0	0	1	0	0	1	1	0	1	1	0	5
Tonsekar et al (2017) (45)	0	1	1	0	1	1	1	1	0	0	0	6
Ungprasert et al (2017) (46)	0	1	1	0	0	1	1	1	1	1	0	7
Xu et al (2017) (47)	0	1	1	0	0	1	1	0	1	1	0	6
Zeng, Leng et al (2016) (48)	0	1	1	0	1	1	0	0	1	1	0	6
Zeng et al (2012) (49)	0	1	1	0	0	1	0	0	1	1	0	5
Zeng, Xia et al (2016) (50)	0	1	1	0	1	1	0	0	1	1	0	6

Abbreviation: AMSTAR, Assessing the Methodological Quality of Systematic Reviews.

## Results

The search strategy was applied on the literature databases PubMed, Embase, Cochrane, and LILACS. We initially identified 1,249 articles; 992 remained after duplicates were removed.

**Figure 1:**
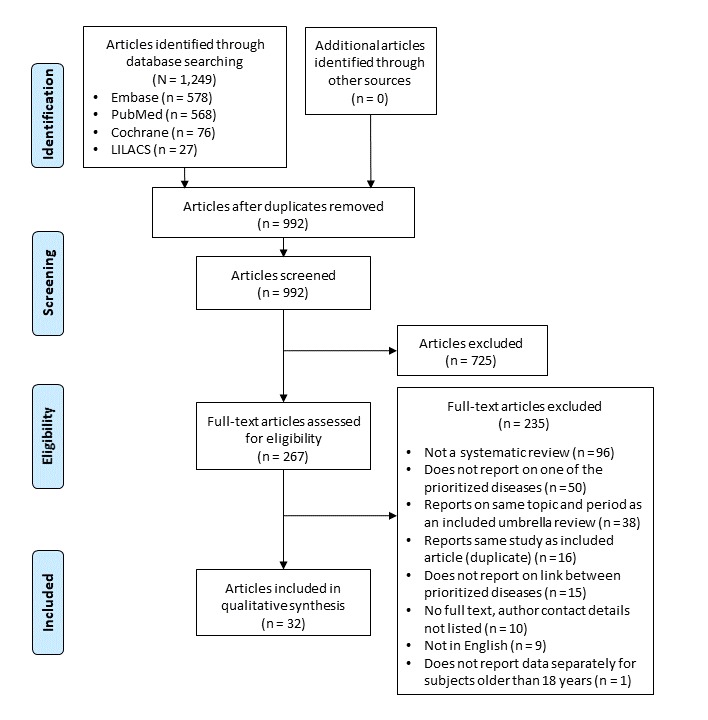
Flow diagram showing exclusion and inclusion process during the literature review based on the Preferred Reporting Items for Systematic Reviews and Meta-Analysis **(**PRISMA) system. Articles were screened for an umbrella review of systematic reviews published between 1995 and 2017 on correlation between prevalent dental conditions and chronic diseases in Germany.

Based on ratings of the 100 first-screened articles, there was good interrater reliability between the 2 reviewers (κ = 0.74). During title and abstract screening, 725 articles were excluded. The remaining 267 articles were evaluated for eligibility in a full-text assessment, and 235 were excluded (Figure 1). Thirty-two studies met the inclusion criteria and were included in the qualitative synthesis (Table 2).

**Table 2:** Characteristics of Included Systematic Reviews, Systematic Umbrella Review of Correlation Between Prevalent Dental Conditions and Chronic Diseases, 1995–2017

Study	Years Searched	Study Type(s)	Population	Chronic Systemic Disease	Dental Disease	Interventions	Outcome	Common Risk Factors/Confounders	Quality Assessment Tool Used	Conclusions
Abdul-jabbar, Javed et al (2017) (19)	Up to March 2016	RCTs	6 Studies, 18–64 patients per study	T2DM	Chronic periodontitis	Laser therapy or antimicrobial photodynamic therapy after SRP	Clinical periodontal outcomes and glycemic outcomes	NA	Jadad	LT alone or aPDT showed significant improvement in the clinical periodontal parameters and glycemic levels in T2DM patients. Future RCTs are warranted to confirm these findings.
Abdul-jabbar, Vohra et al (2017) (20)	Up to October 2016	RCTs	4 Studies, 53–75 patients per study	DM	Chronic periodontitis	aPDT plus SRP/control SRP only	Clinical periodontal outcomes and glycemic outcomes	NA	Jadad	aPDT improved clinical periodontal and glycemic parameters in DM patients. When compared with SRP alone, none of the studies showed additional benefits of aPDT.
Al-Hamoudi (2017) (21)	Up to May 2017	RCTs	6 Studies in Brazil and Saudi Arabia. Number of participants, 20–30; 4 studies of patients with T2DM, 3 studies with cigarette smokers	T2DM	Chronic periodontitis	SRP plus aPDT, (control SRP only)	Clinical (PD reduction and CAL gain): microbiological (bacterial count) and immunological (cytokine profile) outcomes	Smoking	Modified Jadad quality scale for reporting randomized controlled trials	SRP plus aPDT improved clinical periodontal and immunological parameters in T2DM and cigarette smokers, no benefits of aPDT compared with SRP alone.
Azarpazhooh and Leake (2006) (22)	Up to July 2005	Case-control and cross-sectional for COPD	Periodontal disease and COPD: 2 cross-sectional studies and 2 case-control studies; 46 to 13,792 participants	COPD	Periodontal disease, tooth loss (dentulous and edentulous patients): dental plaque	Tooth brushing, decontamination/rinsing	Risk of pneumonia/risk of COPD	NA	NA	Fair evidence of an association of pneumonia with oral health, poor evidence supporting a weak association (OR <2.0) between COPD and oral health, good evidence (I, grade A recommendation) that oropharyngeal decontamination with different antimicrobial interventions reduces the progression or occurrence of respiratory diseases.
Batista et al (2011) (23)	Up to May 2010	Longitudinal, cross-sectional, and case-control studies, measuring PD and atherosclerosis clinically	Longitudinal, cross-sectional, and case-control studies, measuring PD and atherosclerosis clinically	Atherosclerosis	Periodontal disease: measures not standardized	NA	Intima-media thickness (atherosclerosis measure)	See Table 3 per study, no confounders assessed in all studies (mostly age and smoking)	NA	Although most studies reviewed found a positive association between PD and atherosclerosis, methodological limitations raise doubts on the validity. All included studies found a significant association.
Botero et al (2016) (24)	1995 to July 2015	Systematic reviews, with or without meta-analysis	13 Systematic reviews, ranging from 2 studies with 143 participants to 35 studies with 2,565 participants (mostly included RCTs, some also non-RCTs)	DM type 1 and T2DM	Periodontitis	Nonsurgical periodontal treatment, with/without antibiotics (2 studies, flap surgery)	Glycemic control: HbA1c or fasting glucose levels	NA	AMSTAR	Periodontal treatment could help improve glycemic control in patients with T2DM and periodontitis (10/12 systematic reviews with meta-analysis). Whether reduction in HbA1c values (0.23 to 1.03 percentage points) is significant for T2DM treatment and control is unclear. Impact of PT in patients with type 1 diabetes and adjunctive antimicrobials is inconclusive. Eight Reviews were of high quality, 5 moderate, 1 low. Three reviews had low risk of bias, 6 were unclear, and 5 high.
Dai et al (2015) (25)	Up to November 2013	Observational studies (clinical trials were excluded)	23 Observational studies: 6 tooth loss, 4 caries, 3 oral hygiene, 4 periodontal health, with 20−706 patients per study	Stroke	Tooth loss, periodontitis, caries experience	NA	Oral health outcomes and oral health–related behaviors	Oral health behaviors	MORE	Poorer oral health status among patients with a stroke diagnosis compared with healthy controls, greater tooth loss, higher dental caries experience, and poorer periodontal status.
D’Aiuto et al (2017) (26)	2005–2015	Systematic reviews/meta-analyses	30 Systematic reviews: 5–78 studies included per review. Number of participants unclear. Various types of studies included in systematic reviews.	DM	Periodontal disease, tooth loss, caries	NA	Bidirectional relationship, oral health–diabetes	NA	AMSTAR	Strong evidence of T2DM being a risk factor for periodontal diseases, weak evidence in relation to type 1 diabetes. Weak evidence in relation to dental caries experience in children. Limited evidence of periodontitis being a risk factor for diabetes, but evidence of periodontal treatment leading to modest short-term improvement in glycemic control (not sustained beyond 3 months).
D’Aiuto et al (2013) (27)	Up to July 2012	RCT for meta-analysis	14 Studies: 32–160 participants per study	CVD	Periodontal disease	SRP or surgical treatment, tooth extraction, antibiotics	CVD risk factors	Biomarkers subject to methodological and environmental confounders	NA	Main consistent finding after periodontal therapy was a reduction of serum levels of CRP (stable measure of systemic inflammation) and an improvement of measures of endothelial function (which represents a surrogate marker of CVD).
Dietrich et al (2017) (28)	2005–2015	Systematic reviews and/or meta-analyses	22 Systematic reviews. 3–89 studies per systematic review of various types. Number of participants not reported	CVD	Oral health: periodontitis, caries, tooth loss	Oral health promotion, periodontal treatment	NA	NA	AMSTAR and PRISMA	High quality evidence of association between CVD and oral health. Mainly association between chronic periodontitis and atherosclerotic heart disease and is independent of confounding factors. No causal relationship has been established. Firm association between oral health (periodontitis, caries and tooth loss) and atherosclerotic cardiovascular disease, that is, coronary heart disease, stroke, and peripheral vascular disease. Little or no evidence to support any links between oral health and other forms of cardiovascular disease that are non-atherosclerotic such as HT, arrhythmias, and heart failure. Periodontal therapy is associated with reductions in surrogate markers of atherosclerotic CVD.
Faggion et al (2016) (29)	Up to March 2015	Systematic reviews with meta-analysis	11 Meta-analyses, original studies based on 12–514 patients	DM type 1 and T2DM	Periodontal disease	Periodontal treatment	HbA1c levels	NA	AMSTAR and OQAQ	SRs showing an average decrease of 0.46% (median, 0.40%) of HbA1c levels. These values, nevertheless, are not significant when meta-analyses of longer follow-ups (up to 6 mos) are evaluated. Furthermore, most primary studies included in those SRs had several methodological limitations.
Hasuike et al (2017) (30)	Up to July 2015	Systematic reviews with meta-analysis	9 Studies, 60–1,135 participants	DM type 1 and T2DM	Periodontal disease	Periodontal treatment with or without adjunctive use of local drug delivery and systemic antibiotics.	Changes in HbA1c	NA	AMSTAR	Significant effect of periodontal treatment on improvement of HbA1c levels in diabetes patients, although effect size is extremely small. In addition to this small effect size, the supporting evidence cannot be regarded as high quality.
Kelly et al (2013) (31)	Up to May 2012	Systematic reviews	13 Systematic reviews, 9 with meta-analyses. Not reported how many studies were included in each systematic review	Chronic heart disease	Periodontal disease	NA	Quality appraisal	NA	AMSTAR and Glenny et al (51)	Apart from analyzing the methodological and structural quality of the selected systematic reviews and meta-analyses, we did not attempt to perform any outcome analyses. There was substantial heterogeneity in the types of articles included in the 13 reviews, with varying study designs including cohort, cross-sectional, case-control, and RCTs.
Kothari et al (2017) (32)	Through January 2016	Observational studies, case-control studies, and 1 mixed-methods study	27 Studies; no information on number of participants per study	Acquired brain injury, including cerebrovascular diseases	Tooth loss, periodontal status, caries	Professional oral health care or oral hygiene instruction (in some studies)	NA	NA	NA	Currently low level of interest in topic. All included studies reported poor oral health in patients with brain injury. Studies also showed significant improvements in oral health if appropriate measures were implemented at rehabilitation settings. Stroke patients seemed to present with higher incidence of missing teeth and tooth mobility.
Lafon et al (2014) (33)	Up to April 2012	Cohort studies	9 Studies: 5 in North America, started during 1970–1980. Participants ranged from 1,137–51,529. Length of follow-up from 12–57 years	Stroke	Periodontal disease	NA	Periodontitis and tooth loss	NA	Evaluation grid	Results suggested a link between stroke and periodontal diseases. The association was significant for periodontitis and tooth loss. The risk of ischemic or hemorrhagic stroke was higher in people with periodontitis (estimated adjusted risk, 1.63 [1.25–2.00]). Tooth loss was also a significant risk factor for stroke (estimated adjusted risk, 1.39 [1.13–1.65]). In this review, gingivitis did not significantly influence the occurrence of stroke.
Lam et al (2011) (34)	NA	3 RCTs, 3 pre–post interventions, 1 split-mouth, 1 quasi-experimental	8 Studies, ranging from 6–303 patients	CVD	Oral health: periodontal health	Oral health instruction, extractions, periodontal treatment	Periodontal health and changes in systemic blood marker levels	NA	NA	Periodontal interventions were found to be capable of modifying numerous surrogate markers of cardiovascular outcomes including CRP, Ox-LDL, WBC, fibrinogen, IL-6, and endothelial dysfunction. It must be accepted, however, that neither a cause-and-effect relationship, nor the exact mechanism whereby periodontal disease may affect cardiovascular disease risk has been established. Whether the reduction of systemic inflammatory markers can truly decrease the risk of secondary cardiovascular events remains to be shown by studies of longer duration. Interventions aimed at improving periodontal parameters such as plaque and gingival bleeding were successful in patients with HT, CHD, and previous heart transplantation. Periodontal interventions were less successful at effecting changes in CsA-induced gingival overgrowth in heart transplantation patients. None of the effective articles included assessments on the effect of oral promotion interventions on oral microflora.
Leira et al (2017) (35)	Up to March 2015	3 cohort (retrospective and prospective), 5 case-control studies	8 Studies, 95–9,962 patients. Europe, North America, and Asia. Data collected between 1968 and 2012	Ischemic stroke (assessed as acute ischemic lesion on brain imaging and/or neurological deficit, TOAST and ICD)	Periodontitis (assessed with CAL, PPD, and radiographic bone loss)	NA	Risk of ischemic stroke	Most commonly adjusted vascular risk factors were: age, sex, DM, HT, smoking status, hypercholesterolemia, and BMI	GRADE	Suggested a positive association between ischemic stroke and prevalence of periodontitis. The risk of cerebral ischemia was higher in subjects with periodontitis (RR, 2.88 [95% CI, 1.53–5.41]).
Leng et al (2015) (36)	Up to May 2015	Prospective cohort studies	15 Studies enrolling 230–406 participants	Coronary heart disease	Periodontal disease	NA	CHD-related morbidity (fatal and nonfatal) or mortality, evaluated using relative risk or hazard ratio	Sex, BMI, smoking, age, family history of heart disease, education, blood pressure (most common confounders)	NA	Patients with periodontal disease were at a significantly increased risk of developing CHD (RR, 1.19; 95% CI, 1.13–1.26; *P* < .001). Subgroup analyses according to the effect measure, adjustment for confounding factors, median follow-up time, country of study origin, assessment method of periodontal disease, and sex all indicated significant associations between periodontal disease and CHD.
Li et al (2014) (37)	Up to April 2014	RCT and quasi-RCT	1 RCT, 303 participants	CVD	Chronic periodontitis	SRP and community care	Cardiovascular events	NA	Cochrane’s RoB assessment tool, GRADE	The study recorded 12 cardiovascular events, but results were not significant. Also, serum high sensitivity CRP: who had high CRP, and adverse events all reported nonsignificant results. Because only 1 was study eligible for inclusion, which was also judged to be at high risk of bias, the results should be interpreted with caution.
Lira et al (2017) (38)	Up to September 2016	Clinical trials	12 Studies qualitative analysis; 8 meta-analyses, 30–70 patients per study	DM	Periodontal disease	Adjunctive use of systemic antibiotics in nonsurgical periodontal treatment, compared with nonsurgical periodontal treatment alone.	Changes in HbA1c	NA	Cochrane’s RoB assessment tool	Shows no additional benefit of associating systemic antibiotics to nonsurgical periodontal treatment versus SRP alone in improving HbA1c levels 3–4 months after treatment.
Martin-Cabezas et al (2016) (39)	2000 to June 2016	Longitudinal studies or case-control studies and cross-sectional studies	25 Studies in review; 18 in meta-analysis: 20 cross-sectional, 3 case-control, and 2 longitudinal studies, across Asia, Europe, United States, and Africa. Ranging from 8,124–1,025,340 participants.	HT	Periodontal disease	NA	HT	Age, sex, smoking, BMI, binge drinking	NOS	Results from the present meta-analysis support the association between HT and periodontal diseases with a range of ORs from 1.15 to 1.67. Highest OR was calculated when severe form of periodontitis with secure diagnosis criteria was considered (OR, 1.64).
Mauri-Obradors et al (2017) (40)	1998 to January 2016	Primary studies	19 Studies: 4× longitudinal studies; 15× cross-sectional studies. A total of 3,712 patients, of whom 2,084 had diabetes.	DM type 1 and T2DM	Caries, periodontal disease, BMS, oral mucosa alterations	NA	Oral manifestations	NA	Recommendations made by OCEBM	DM leads to multiple complications, which increase when glycemic control of the patient is inadequate. The main oral complication attributed to diabetes is periodontal disease: considered the sixth complication of DM. Higher prevalence of periapical lesions in patients with poorly controlled diabetes. Information presented in the literature about the relationship between the DM and tooth decay is inconsistent.
Orlandi et al (2014) (41)	Through January 2014	Cross-sectional studies, case-control studies, population surveys, cohort studies, pilot studies, controlled trials, RCTs	35 Studies for systematic review, 22 studies for meta-analysis; 2,021 cases, 3,431 control	c-IMT; FMD	Periodontitis	Periodontal intervention	Increase in c-IMT. Effects of periodontal treatment on FMD.	CVD (age, sex, systolic blood pressure, HDL-C, smoking, diabetes, HT treatment, and total cholesterol). Athero-sclerosis	Newcastle-Ottawa Quality Assessment Scale	Diagnosis of PD was associated with a mean increase in c-IMT of 0.08 mm (95% CI, 0.07–0.09 mm) and a mean difference in FMD of 5.1% compared with controls (95% CI, 2.08%–8.11%). A meta-analysis of the effects of periodontal treatment on FMD showed a mean improvement of 6.64% between test and control (95% CI, 2.83%–10.44%). Periodontal disease is associated with greater subclinical atherosclerosis as assessed by increased c-IMT and an independent predictor of cardiovascular events in high-risk populations. There is evidence of an impaired FMD, which is restored by periodontal treatment in individuals having periodontal disease.
Sanchez et al (2017) (42)	NA	3 MA/SR of RCT, 1 MA/SR of RCT and single cohort studies, 1 SR of oral health promotion interventions, 1x SR of RCT/quasi-RCT, 1 MA/SR, 1 MA/SR of intervention trials, 1 MA of pilot trials, 1 MA/SR of intervention and nonintervention trials, SR of intervention trials; 2 SR, 1 LR, 1x pre–post mixed design, 1 pilot of an oral health program, 1 oral health guidelines for prenatal care, 1x best practice recommendations; 1 RCT, 1 pre–post test design, 1 pilot of an education program, 1 pre–post mixed design, 1 pilot of an oral health education model, 2 cross-sectional studies, 3 pilots of a screening tool, 1 best practice recommendations	34 Studies included from Australia, Europe, United States, France, Italy, United Kingdom, Turkey, Sweden, England	CVD	Periodontal disease	Periodontal treatment	CVD	NA	AMSTAR	Strong association between periodontal disease and CVD. Although a causal link has not been confirmed between periodontal disease and CVD, the general consensus is that cardiovascular patients need to be made aware of this association and its potential implications.
Schmitt et al (2015) (43)	Up to September 2014	RCTs: case-control studies, cross-sectional studies, prospective cohort pilot study	Studies included in qualitative synthesis = 10; studies included in quantitative synthesis =7; sample size in total 2,257 (range, 26–814)	Arterial stiffness	Periodontitis	Periodontal treatment	Primary outcome had to be the measure of arterial stiffness by means of pulse wave velocity assessment.	Age, sex, smoking, or diabetes	GRADE system	The present systematic review and meta-analysis support an association between severe periodontitis and increased pulse wave velocity. The measurement of arterial stiffness provides a cardiovascular marker of the cumulative impact of both known and unknown risk factors, which may include periodontitis.
Teeuw et al (2014) (44)	Up to June 2013	RCTs, CCTs	Studies included n = 20; cases in total n = 865 (11–212 patients per study)/control in total n = 657 (11–105 patients per study). Cases and control in total n = 1522.	Atherosclerosis	Periodontitis	Treatment of periodontitis	Clinical CVD parameters (ie, clinical event, such as angina pectoris, MI, stroke, death) and/or markers related to atherosclerosis and CVD risk, including markers of systemic inflammation and thrombosis, lipid and glucose metabolism, and vascular function.	Overweight and smoking	GRADE	PT reduces the risk for CVD by improving plasma levels of inflammatory (CRP, IL-6, TNF-a), thrombotic (fibrinogen), and metabolic (triglycerides, TC, HDL-C, HbA1c) markers and endothelial function. This improvement is sustained well more than 6 months after therapy, and it is greater in those individuals having both periodontitis and co-morbidities like CVD and/or DM. Our findings emphasize the effectiveness and need for periodontal diagnosis and periodontal therapy in atherosclerotic and diabetic individuals to improve their systemic health.
Tonsekar et al (2017) (45)	Up to April 2016	4x retrospective cohort, 3x prospective cohort, 1x case-control study nested in a longitudinal study	Studies included n = 8; 4,075 participants; number of participants 144 to 911; countries: United States, South Korea, France, Sweden.	Dementia	Periodontal disease, tooth loss	NA	Outcome measured was assessed by verified cognitive tests such as Mini-Mental State Examination: Delayed Word Recall and Digit Symbol Substitution Test.	Apolipoprotein E (ApoE) allele, considered a major genetic risk factor for Alzheimer disease and a possible confounding factor in the association between periodontitis and dementia.	Newcastle-Ottawa Scale	Association between subsequent dementia, periodontal disease and tooth loss was inconclusive.
Ungprasert et al (2017) (46)	Up to July 2016	Case-control or cohort study	Studies included n = 5; number of subjects (cases/comparators) 1) 115,365/115,365; 2) 1,358/70,020; 3) 100/100; 4) 50/121; 5) 60/45. The 5 studies included 312,584 subjects. Countries: Taiwan, United States, Greece, Norway, Italy.	Psoriasis	Periodontitis	NA	Periodontitis and risk of psoriasis	Confounders: smoking, obesity, and DM	Newcastle–Ottawa quality assessment scale	Patients with periodontitis have a significantly increased risk of psoriasis.
Xu et al (2017) (47)	Up to July 2016	6x cross-sectional, 12x case control, 4x cohort studies	Studies included n = 22; 129,630 participants; countries: United States, Sweden, Japan, India, Spain, Iran, China, Germany, Greece.	MI	Periodontal disease	NA	Periodontal disease (including pocket probing depth, attachment loss, bleeding on probing, plaque index, gingival index, X-ray, and microbiological results) and the risk of myocardial infarction	Risk factors including age, smoking, and diabetes are common in both PD and MI	Newcastle-Ottawa Scale	Significant association between periodontal disease and MI. Subgroup analyses also confirmed the elevated risk for MI in periodontal disease subjects.
Zeng, Leng et al (2016) (48)	Up to February 20, 2015	10x cross-sectional, 5x case control	Studies included n = 15; 17,330 participants; countries: United States, Sweden, Germany, Austria, Italy, Spain, Japan, Portugal, Poland, South Korea, China.	Carotid atherosclerosis	Periodontal disease	NA	Risk of carotid atherosclerosis as diagnosed by c-IMT (by ultrasound) or carotid plaque thickness (by panoramic radiographs)	Common risk factor: smoking; confounder: DM	NA	Periodontal disease was associated with carotid atherosclerosis, although available evidence is insufficient to confirm the causal relationship of periodontal disease and carotid atherosclerosis.
Zeng et al (2012) (49)	Up to January 10, 2012	Observational studies (cross-sectional, case-control, or cohort design)	Studies included n = 14; subjects (case/control): between 28/30 and 810/12,982. Countries: United States, Poland, Norway, Iran, China, India.	COPD	Periodontal disease	NA	Relationship between PD and COPD	NA	NA	Periodontal disease significantly increases the risk of COPD, with the increase being likely independent of conventional COPD risk factors. Dental plaque that contains bacteria may be responsible for COPD, therefore, good attention to teeth brushing and general oral hygiene care may reduce the risk of COPD.
Zeng, Xia et al (2016) (50)	Up to June 10, 2015	Cohort and nested case-control studies	Studies included n = 5; subjects: (lung cancer/sample): 1)191/11,328; 2)236/48,375; 3) 225/30,666; 4) 243/153,566; 5) 754/77,485. Countries: United States, Sweden, China.	Lung Cancer	Periodontal disease	NA	Risk of lung cancer in patients with periodontal disease	Smoking	NA	Periodontal disease is associated with a significant and increased risk of lung cancer.

Abbreviations: AMSTAR, Assessing the Methodological Quality of Systematic Reviews; aPDT, antimicrobial PhotoDynamic Therapy; BMI, body mass index; BMS, burning mouth syndrome; CAL, clinical attachment level (14); CAL, clinical attachment loss (29); CCT, controlled clinical trial; CHD, coronary heart disease; CI, confidence interval; c-IMT, carotid intima-media thickness; COPD, chronic obstructive pulmonary disease; CRP, C-reactive protein; CsA, cyclosporin A; CVD, cardiovascular disease; DM, diabetes mellitus; FMD, flow-mediated dilation; GRADE Grading of Recommendations, Assessment, Development and Evaluations; HbA1c, glycated hemoglobin; HDL-C, high-density lipoprotein cholesterol; HT, hypertension; ICD, International Classification of Diseases; IL, interleukin; LT, laser therapy; MA, meta-analysis; MI, myocardial infarction; MORE, Methodological Evaluation of Observational Research; NA, not applicable; NOS, Newcastle-Ottowa Scale; OCEBM, Centre for Evidence-Based Medicine, Oxford; OQAQ, Overview Quality Assessment Questionnaire; PD, probing depth; PPD, probing pocket depth; PT, periodontal therapy; PRISMA, Preferred Reporting Items for Systematic Reviews and Meta-Analyses; OR, odds ratio; Ox-LDL, oxidized low-density lipoprotein; RCT, randomized controlled trial; SR, systematic review; SRP, scaling and root planing; T2DM, type 2 diabetes mellitus; TC, total cholesterol; TOAST, Trial of Org 10172 in Acute Stroke Treatment; WBC, white blood cell.

### Methodologic quality of systematic reviews

The quality of all SRs included in the qualitative synthesis was assessed using the 11-point AMSTAR checklist (Table 1). In our assessment, SRs met between 3 and 10 of the possible 11 criteria (median = 6). No review complied with all 11 points of the tool.

Criterion 3 (“Was a comprehensive literature search performed?” [n = 32]) and criterion 6 (“Were the characteristics of the included studies provided?” [n = 31]) were met by nearly every SR. Criterion 11 (“Was the conflict of interest included?” [n = 5]) was rarely met. Criterion 7 (“Was the scientific quality of the included studies assessed and documented?” [n = 23]) and criterion 10 (“Was the likelihood of publication bias assessed?” [n = 12]) were fulfilled by many of the studies. Two studies were determined to be low quality, 26 studies were moderate quality, and 4 studies were high quality.

### Characteristics of included SRs

The primary studies included in the SRs were conducted between 1995 (24) and May 2017 (21) (Table 2). The included SRs varied in diverse aspects. Multiple primary studies, including randomized controlled trials (RCTs) (14,15), case-control studies (CCSs) (22,23), cross-sectional studies (22,23), cohort studies (22), clinical trials (25), observational studies (32), mixed-method studies (32), pilot studies (41), and population surveys (41) were examined. The primary studies differed by study population, from 303 participants in an RCT (37) to 1,025,340 subjects in a CCS (39). They also differed by location; studies were conducted in Europe (Austria, Belgium, France, Germany, Greece, Italy, Norway, Poland, Portugal, Spain, Sweden), North America (United States), South America (Brazil), and Asia (China, Iran, Japan, Saudi Arabia, South Korea, Taiwan).

Fifteen different disease combinations were examined in the included SRs (Table 3). Multiple studies reported on common risk factors that can have a progressive effect on dental and chronic systemic conditions. The most frequently mentioned were smoking (21,23,35,36,39,41,43,44,46–48,50), age (23,35,36,39,41,43,47), sex (35,36,39,41,43), and body mass index (BMI) or overweight (35,36,39,44,46).

**Table 3:** Number of Systematic Reviews Observing Disease Correlations, Systematic Umbrella Review of Correlation Between Prevalent Dental Conditions and Chronic Diseases, 1995–2017^a^

Dental or Chronic Disease	Periodontitis	Tooth Loss	Dental Caries	Ʃ
Diabetes mellitus	46 (41/5)	1 (1/0)	4 (1/3)	51
Cardiovascular disease	33 (22/11)	6 (6/0)	2 (1/1)	41
Cerebrovascular disease	4 (0/4)	2 (0/2)	2 (0/2)	8
Chronic obstructive pulmonary disease	2 (0/2)	1 (0/1)	—	3
Dementia	1 (0/1)	1 (0/1)	—	2
Psoriasis	1 (0/1)	—	—	1
Lung cancer	1 (0/1)	—	—	1
**Total**	**88**	**11**	**8**	**107**

Abbreviation: — , not applicable.

a The first number in the parentheses indicates the number of systematic reviews included in the umbrella review; the second number indicates the number of reviews that were individually included in the systematic reviews.

In addition to reporting on common risk factors, multiple studies reported on chronic systemic diseases increasing the risk of developing a dental condition and vice versa. D’Aiuto et al (26) reported strong evidence for T2DM being a risk factor for periodontal diseases. Leng et al (36) reported that patients with a periodontal disease have a significantly increased risk for developing coronary heart disease, and patients with periodontitis have an elevated risk for myocardial infarction (47). Multiple studies reported on associations between cerebrovascular diseases (eg, stroke) and dental conditions. For example, Lafon et al (33) reported that the risk of ischemic or hemorrhagic stroke was higher for people with periodontitis (estimated adjusted risk, 1.63 [95% confidence interval (CI),1.25–2.00]) and that tooth loss is a significant risk factor for stroke (estimated adjusted risk, 1.39 [95% CI, 1.13–1.65]). Likewise, Leira et al (35) found that the risk of cerebral ischemia was higher in subjects with periodontitis (relative risk, 2.88 [95% CI, 1.53–5.41]), suggesting a positive association between ischemic stroke and the prevalence of periodontitis. Another study reported that periodontal disease significantly increases the risk of COPD (49).

### Summary of the systematic reviews

The studies included in the analysis reported on 107 correlations between dental conditions and chronic systemic diseases. Among the 32 SRs included in the qualitative synthesis, 6 were umbrella reviews. These 6 umbrella reviews incorporated 98 SRs, but 2 of the umbrella reviews investigated multiple disease correlations, not all of which met the inclusion criteria of this review. Therefore, in the analysis of disease correlations, 107 SRs were included.

The most frequently observed dental condition that was correlated with chronic systemic diseases was periodontitis (n = 88). Links between tooth loss and chronic systemic diseases (n = 11) and dental caries with chronic systemic diseases (n = 8) were observed less often.

In terms of chronic systemic diseases, most correlations with dental conditions were identified for T2DM (n = 51) and CVD (n = 41). Less frequently observed were correlations with cerebrovascular disease (n = 8), COPD (n = 3), dementia (n = 2), psoriasis (n = 1), and lung cancer (n = 1).

Most disease correlations were found for periodontitis with T2DM (n = 46) (19–21,24,26,29,30,38,40) and periodontitis with CVD (n = 33) (23,27,28,31,34,36,37,39,41–44,47,48). This was followed by SRs indicating correlations of tooth loss with CVD (n = 6) (28), periodontitis with cerebrovascular disease (n = 4) (25,28,32,35), and dental caries with T2DM (n = 4) (26). For the remaining diseases, between 0 and 2 correlations were observed.

**Figure 2:**
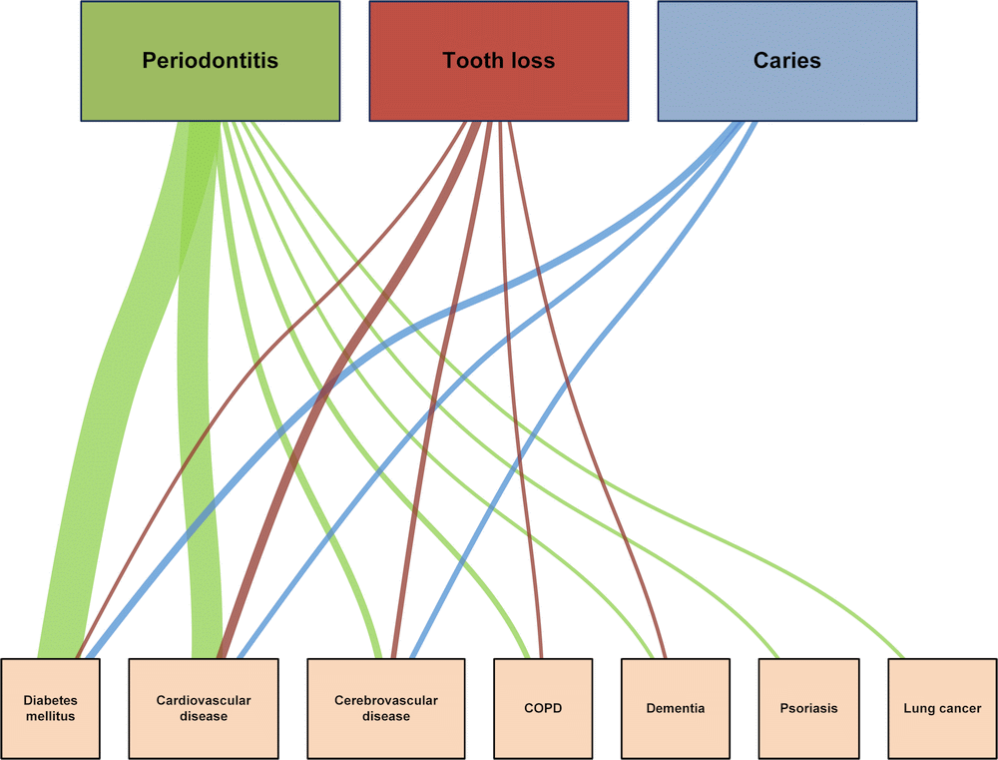
Illustration of the number of identified systematic reviews that showed disease correlations, umbrella review of systematic reviews published between 1995 and 2017 on correlation between prevalent dental conditions and chronic diseases in Germany. Width of lines illustrates the number of systematic reviews that report on the disease combinations. Abbreviation: COPD, chronic obstructive pulmonary disease.

The results of the data extraction showed that the included SRs indicated that there was an absence of causal evidence between the reported diseases. This was reported for correlations of CVD with periodontitis (42,48) and cerebrovascular disease with dental caries (29). None of the included SRs, which reported on links between periodontitis and diabetes mellitus, reported to have specifically investigated about causal inference concerning the examined diseases (Figure 2).

## Discussion

In our umbrella review, we found that of all the interrelationships between dental conditions and chronic systemic diseases described in the included systematic reviews, periodontitis was the dental condition with the most reported correlations to chronic systemic diseases and T2DM was the chronic condition for which most correlations to dental conditions were found. The most frequently reported correlations were 1) periodontitis with T2DM and 2) periodontitis with CVD. 

The identified correlations should be carefully considered in the care provided to multimorbid patients with combinations of dental conditions and chronic systemic diseases. These patients may potentially benefit from an increased sensibility and awareness of practitioners for disease correlations, the potential for earlier diagnosis, and better coordination of the attending physicians. In this context, our findings can support practitioners by highlighting correlating diseases through common risk factors (eg, smoking) and disease indicators (eg, high hemoglobin A1c). For example, dentists treating patients with difficulties in controlling chronic periodontitis should consider the possibility of correlating chronic systemic conditions that worsen recovery and accelerate recurrence, such as T2DM. By coordinating the treatment with the attending physician or diabetes specialist, treatment and control of both correlating diseases can be improved. Better integration of diabetes and periodontal care has also been highlighted in international medical guidelines (52,53). Further improvement of intersectoral care necessitates that both dentist and general practitioner are sufficiently aware of existing correlations between dental conditions and chronic systemic diseases and how these correlations may influence treatments. For the treatment of diseases that are linked but treated by separate groups of health care professionals, communication, information exchange, and decision support can contribute to greater quality of care. At the same time, unnecessary medical interventions should be avoided if there is no solid evidence base supporting a possible benefit for the patient.

As for the correlation of periodontitis with T2DM, our findings indicate substantial evidence. In addition, the included studies suggest that the treatment of periodontitis may improve the glycemic regulation of T2DM patients (19,20,24,26,29,30). Although the association between periodontitis and T2DM was most frequently studied among the included SRs, the SRs did not report to have specifically investigated about causal inference concerning the relationship between both diseases. Conversely, all SRs that investigated causality between dental conditions and other chronic diseases reported congruently about insufficient evidence to determine causality. As a result, we could not ultimately confirm that the identified relationships are causal.

For 2 disease correlations, periodontitis with T2DM and periodontitis with CVD, the existence of a correlation could be confirmed by multiple SRs. In case of other disease correlations (tooth loss with CVD, dental caries with DM, and periodontitis with cerebrovascular disease), evidence was present for only a few reviews (n = 4–6). There was evidence of a correlation for the remaining conditions, although it was limited (n = 1–2), and the existing evidence is still unclear. Regardless of the level of evidence for any of the correlations, the conclusiveness of currently existing evidence often remains vague. In some cases, studies contradicted or differed from each other with regard to the assessment.

When assessing potential causal pathways between dental conditions and chronic systemic diseases, common risk factors play an important role. They can have a direct or indirect impact on multiple disease entities. The SRs frequently reported common risk factors for dental and chronic systemic conditions, including smoking, age, sex, and BMI/overweight. A study by Sheiham and Watt (54) reported additionally about diet, hygiene, alcohol use, stress, and trauma as important common risk factors. Because common risk factors increase the possibility of further diseases in chronically ill patients, they can be used as indicators for the development or presence of another related disease. Raising health care practitioners’ awareness of this issue may improve the prevention and early detection of comorbidities for chronically ill patients. In the context of intersectoral patient care, common risk factors should be considered to identify patients who should be referred to another specialist to verify a suspected comorbidity. Patients with comorbidities in particular could benefit from a better cooperation and coordination among the attending practitioners in various disciplines (7).

The study has several limitations. First, because of the heterogeneous quality of the included SRs, the evidence on links between chronic systemic and dental conditions should be interpreted with caution. However, to counteract the risk of bias by including heterogeneous and low-quality SRs, we assessed the quality of the reviews with the AMSTAR (18) tool, and the evaluation showed that the heterogeneity was moderate: 2 reviews were low quality, 26 were moderate quality, and 4 were high quality. In addition, the large number of included studies necessitated a more general overview than would be possible in a study focusing on specific diseases. However, this umbrella review was designed to summarize existing knowledge for links between dental conditions and chronic systemic diseases from a broad perspective. Because we used a broad search strategy, our search may not have identified studies using definitions that are not common in literature. In order not to miss any relevant SR or disease in spite of the broad search strategy, we included the most commonly used terms for each of the focused diseases, including key terms and categorizations used in each database. Medical terms that are often hidden under various classifications and definitions (eg, periodontitis [55]: chronic periodontitis, periodontosis, aggressive periodontitis, periodontal disease) were included, and the search was checked by 2 experts to ensure that all relevant terms were included.

Second, the included SRs documented various disease correlations, including different types of studies, populations, interventions, and outcomes. This, and differences in the research questions of the included SRs, restricted the comparability of our results. This showcases a high degree of heterogeneity in the literature on chronic-dental disease links. For example, numerous definitions and biomarkers for periodontitis have been used in the literature, and this may affect any overview of studies reporting on correlations between periodontal and chronic systemic diseases. Third, given the variety of chronic systemic diseases and the specific context for which this study was conducted, we prioritized chronic systemic diseases according to the prevalence of disease in Germany. Therefore, our findings may not be generalizable to other settings or contexts. We set this priority because the ultimate objective of this project (8) is to apply our findings to German routine care and to improve multimorbid patient care by general practitioners and dentists. But because the burden of disease in Germany is similar to that of other Western European countries (10) and because the consideration and treatment of patients with dental conditions and general diseases is analogous worldwide, our findings are more broadly transferable.

Despite the limitations, to our knowledge our study is the first that provides a systematic and comprehensive overview and quality assessment of the evidence on correlations between highly prevalent dental conditions and chronic diseases, as reported in previously published SRs. Given the worldwide high prevalence and incidence of dental conditions and increasing co-occurrence with chronic systemic diseases, our findings are relevant and raise awareness for potential opportunities of better integrating medical and dental care.

### Future research direction

The presented overview of correlations between dental conditions and chronic systemic diseases could be used as a guide to prioritize future studies on disease interdependencies, with particular attention being given to making causal inference. Focus should be set on the identification of the best-substantiated correlations and gaps in the study of disease correlations. To reduce uncertainties and to adequately raise awareness for disease correlations, it is important to provide health care practitioners and patients with information about the extent to which there is decisive evidence with respect to (potentially) causal disease links. For this purpose, clinical guidelines for intersectoral care could improve patient care. Yet, in the absence of robust and decisive evidence, guideline development continues to be highly challenging. In addition, even when guidelines can be developed, serious concerns have been raised about the persistence of “implementation gaps” (7,56). To promote the development of intersectoral guidelines and provide practitioners with fundamental knowledge about disease correlations, research should focus on the underlying causes and extent of disease relationships. Furthermore, it should be assessed how and to what extent interventions can support the treatment and prevention of correlating diseases. Research into the causality underlying disease correlations is an important basis for guiding interdisciplinary collaboration and development of guidelines.

Not least, another promising opportunity to improve the translation from knowledge into action is the development of electronic decision support systems, such as the initiatives conducted by the Agency for Healthcare Research and Quality (57). Thereby, to promote joint considerations of practitioners who treat patients with comorbid conditions, it is also important to decipher the role of common risk factors, which may serve as early markers to initiate pathways of intersectoral care.

### Conclusion

This review contributes to the literature by comprehensively summarizing the evidence, identifying and evaluating the most frequently reported disease correlations and common risk factors, and aggregating the information to provide information about the extent to which there is decisive evidence with respect to the design of intersectoral care processes. The most frequently reported correlations were found for periodontitis with diabetes mellitus type 2 and for periodontitis with cardiovascular disease. Associated common risk factors were smoking, age, sex and overweight. Correlations between dental and chronic systemic diseases have frequently been reported but the existing evidence remains unclear with respect to causal inference. Future research should therefore focus on the causality of disease links in order to provide more decisive evidence with respect to the design of intersectoral care processes. More decisive evidence would also be relevant for future prioritization in the design of intersectoral care processes and the development of electronic decision support systems. 
